# Clinical Uses and Short-Term Safety Profile of Ethiodized Poppy Seed Oil Contrast Agent in the Diagnosis and Treatment of Vascular Anomalies and Tumors

**DOI:** 10.3390/diagnostics11101776

**Published:** 2021-09-27

**Authors:** Robert K. Clemens, Tim Sebastian, Cindy Kerr, Ahmad I. Alomari

**Affiliations:** 1 Vascular Center, Cantonal Hospital Baden, CH-5404 Baden, Switzerland; 2 Department of Radiology and Vascular Anomalies Center, Boston Children’s Hospital and Harvard Medical School, Boston, MA 02115, USA; cindy.kerr@childrens.harvard.edu (C.K.); ahmad.alomari@childrens.harvard.edu (A.I.A.); 3 Clinic for Angiology, University Hospital Zurich and University Zurich, CH-8091 Zurich, Switzerland; tim.sebastian@usz.ch

**Keywords:** ethiodized poppy seed oil, EPO, vascular malformation, sclerotherapy, embolization, safety

## Abstract

Background: There is a sparsity of data on the use of ethiodized poppy seed oil (EPO) contrast agent (Lipiodol) in patients. We investigated the safety of EPO in children, adolescents, and some adults for diagnostic and therapeutic interventions. Methods: All patients who underwent procedures with EPO between 1995 and 2014 were retrospectively included. Demographic characteristics, diagnosis, dose, route of administration, preparation of EPO in combination with other agents, and complications were recorded. Results: In 1422 procedures, EPO was used for diagnostic or treatment purposes performed in 683 patients. The mean patient age was 13.4 years (range: 2 months–50 years); 58% of patients were female. Venous malformations (*n* = 402, 58.9%) and arteriovenous malformations (*n* = 60, 8.8%) were the most common diagnosis. Combined vascular anomalies included capillary–lymphatic–venous malformations, fibroadipose vascular anomalies (*n* = 54, 7.9%), central conducting lymphatic anomalies (*n* = 31, 4.5%), lymphatic malformations (*n* = 24, 3.5%), aneurysmal bone cysts (*n* = 22, 3.2%), and vascularized tumors (*n* = 11, 1.6%). In 1384 procedures (96%), EPO was used in various combinations with sclerosing and embolization agents, including sodium tetradecyl sulfate, ethanol, and glue. The mean volume of EPO used in interventions was 3.85 mL (range: 0.1–25 mL) per procedure with a mean patient weight of 45.9 kg (range: 3.7–122.6 kg) and a weight-adjusted dose of 0.12 mL/kg (range: 0.001–1.73 mL/kg). In 56 procedures (4%), EPO was used as a single agent for diagnostic lymphangiography. The mean volume was 4.8 mL (range: 0.3–13 mL) per procedure with a mean patient weight of 27.4 kg (range: 2.4–79.3 kg) and a weight-adjusted dose of 0.2 mL/kg (range: 0.04–0.54 mL/kg). Procedural-related complications occurred in 25 (1.8%) procedures. The 20 minor and 5 major complications were related to the primary treatment agents. None of them were directly related to EPO. No allergic reactions were noted. Conclusion: The use of an ethiodized poppy seed oil contrast agent in children, adolescents, and adults for diagnostic or therapeutic purposes is safe.

## 1. Introduction

Ethiodized poppy seed oil (EPO) has been used for various diagnostic and therapeutic purposes for over a century [1,2]. Lipiodol (Lipiodol Ultrafluide, Laboratoire Guerbet, Aulnay-Sous-Bois, France), which was approved by the FDA in 1954, is currently the only FDA-approved oil-based contrast agent in the US market following the discontinuation of Ethiodol (Savage Laboratories, Melville, NY, USA) in 2010. Ethiodized poppy seed oil contrast agent is supplied as clear, pale yellow to amber-colored oil in sterile 10 mL vials. The viscous liquid contains 480 mg/mL of iodine organically combined with ethyl esters of fatty acids of poppy seed oil. At 20 °C, the viscosity of Lipiodol ranges between 34 and 70 cP and has a density of 1.28 g/mL 17, compared to 1.0 cP and 1.0 g/mL for water, respectively [3]. Due to its high viscosity and dense radiopacity, approved indications for the use of EPO include lymphangiography, hysterosalpingography, and intra-arterial imaging/treatment of hepatocellular carcinoma [4,5,6]. EPO is considered a standard treatment for unexplained and endometriosis-related infertility [7,8], but the dosage of EPO should be as low as possible to minimize the risk of fetal or neonatal thyroid dysfunction [9]. It has also been widely used in dacryocystography for many decades [10]. In rural countries with a high prevalence of endemic goiter due to iodine deficiency, EPO injections show a beneficial effect on thyroid function in children [11]. Other uses of EPO in children, such as in the diagnosis of malabsorption and for bronchography, have been abandoned [12,13].

The use of injectable EPO is generally safe. Nevertheless, several complications have been reported, including pulmonary embolism, transient fever, lymphangitis, transient hypothyroidism, allergic reaction, local inflammation, foreign body reactions, and paradoxical emboli causing stroke [4,5,6,14,15,16,17,18]. Due to its high viscosity, EPO can cause temporary occlusion of the vessel and ischemia, particularly the pulmonary artery or systemic arteries, in the presence of right-to-left shunts [19].

At our pediatric institution, EPO has been used for the preparation of sclerosing and embolic agents and lymphangiography for decades.

The published data on the feasibility, safety, and types of EPO used during interventions, particularly in children, remains limited. A few reports described the use of EPO and potential complications for lymphangiography and interventions for vascular malformations in children [20,21,22,23].

The aim of this retrospective study was to analyze a single institution’s experience in using EPO for diagnostic and therapeutic indications, techniques, doses, and safety in a large cohort of children, adolescents, and some adult patients.

## 2. Materials and Methods

Study Design, Methods, and Data collection: the institutional review board of the Boston Children’s Hospital, Massachussetts, USA, on clinical investigation approved this retrospective study that was conducted according to the ethical guidelines of the Declaration of Helsinki (IRB-P00005202) in March 2014. Medical records were reviewed for patients who underwent interventional procedures in which EPO was used between August 1995 and August 2014 (18 years). Data collected included age, gender, diagnosis, weight, anatomical location, and type, as well as number of procedures. For each procedure, the dose and route of administration of EPO and treatment agents as well as complications were collected. All procedures utilized sterile techniques, and the vast majority of cases were performed under general anesthesia.

Sclerotherapy refers to the treatment of slow-flow malformation, and embolization refers to the treatment of high-flow malformation and hypervascular lesions regardless of the agents used.

Preparation of embolic and sclerosing agents: N-butyl cyanoacrylate (NBCA) tissue adhesives were utilized for embolization of high-flow lesions (e.g., arteriovenous malformations and tumors) and deep venous malformations: Histoacryl (B. Braun, Melsungen, Germany) and Trufill (Cordis Neurovascular, Miami, FL). NBCA was prepared with EPO at various concentrations (ratio of 3:1 yielding 25% NBCA concentration). Tungsten powder was occasionally used for opacification. Dehydrated ethanol (American Regent, Inc. Shirley, NY) was used for the treatment of venous and arteriovenous malformations. Ethanol was opacified with small volumes of EPO (5:1 ratio) or less commonly with metrizamide powder, which has been discontinued. Foam preparation of the detergent sclerosant 3% sodium tetradecyl sulfate (3% STS) (Sotradecol, Mylan Institutional, Galway, Ireland or Fibrovein, STD Pharmaceuticals, Hereford, UK) was used for sclerotherapy of venous malformations or, less commonly, lymphatic malformations. The stability of the foam made of 3% STS was frequently enhanced by a small volume of EPO (STS to EPO of 5:1) before suspending it in an equal volume of air via a three-way stopcock.

For lymphangiography, EPO was used in conventional pedal technique before 2011 [24] and for intranodal lymphangiography thereafter as described by Rajebi et al. [25]. EPO dosage: when used in hysterosalpingography, the amount of EPO should not exceed 15 mL, and especially pregnancy, uterine bleeding and endocervicitis should be ruled out beforehand. In selective hepatic intra-arterial injection, the dosage should not exceed 20 mL. In lymphography, EPO is injected into a lymphatic vessel under radiologic guidance. For unilateral lymphography of the upper extremities, an amount of 2–4 mL of EPO is recommended, while 6–8 mL can be used in unilateral lymphography of the lower extremities 6–8 mL. Other indications include penile lymphography (2–3 mL of EPO recommended) or cervical lymphography (1–2 mL recommended). In pediatric patients, the amount of 0.25 mL/kg should not be exceeded [26]. The maximal dose of EPO was typically limited to 0.25 mL/kg or <25 mL per procedure.

Any complications that occurred during the intervention and immediate postoperative period, including the post-anesthesia recovery unit, were documented and categorized according to the Society of Interventional Radiology (SIR) classification system for complications by outcome [27], taking into consideration complications known to be caused by the primary sclerosants. Temporally events can be linked directly to specific agents. Expected changes following treatment are swelling, pain, or hemoglobinuria following sclerotherapy [28]. Adverse events are often anesthesia-related. Trivial extravasation, swelling of lymph nodes after EPO injection, and minor bleeding at access sides are not considered as potential adverse events of EPO. Statistical analyses were created in Excel (Microsoft Corporation, Redmond, WA). Descriptive statistics are presented as means (SD) for parametric data and medians (range) for nonparametric data, and categorical variables are presented as frequencies (percentage).

Strength and limitations of this study: the strength of this study is that the data comprised a large number of procedures in which EPO was used for various indications over a long period of time.

This retrospective review has several limitations. Detailed follow-up was not available for all patients. Older records did not show the exact dose of EPO used. It is also important to note that this report is based on information collected in the periprocedural period and not on comprehensive clinical notes. The outcomes in this study may have been influenced by major confounding factors. EPO was mainly used in combination with other agents that may have influenced the outcome. It is difficult to clearly ascertain whether the reported complications were caused by the sclerosing/embolic agents or by the EPO itself. The exact nature of the interactions between EPO and these agents has not been elucidated yet. There was no laboratory testing performed to determine the blood level of the EPO or any of its components. Only the adverse events documented in the immediate postoperative period were culled. Only data for the short-term use and safety profile of EPO in children and adolescents are presented. Data on adults are limited and do not include procedures such as hysterosalpingography or TACE in hepatocellular carcinomas, amongst others, as this cohort is highly selective, consisting of patients with vascular anomalies and tumors. This study does not give valid information on the long-term safety, for example, in lymphangiography, where EPO can persist for years, potentially causing cross-allergies and scar formation.

## 3. Results

The most common diagnosis documented in 402 patients (58.9%) was venous malformation (both sporadic and inherited types), followed by arteriovenous malformations in 60 patients (8.8%). A total of 54 patients (7.9%) had combined vascular anomalies, including capillary–lymphatic–venous malformation and fibroadipose vascular anomaly. Less common diagnoses included central conducting lymphatic anomalies (*n* = 31, 4.5%), lymphatic malformation (*n* = 24, 3.5%), aneurysmal bone cyst (*n* = 22, 3.2%), vascularized tumors (*n* = 11, 1.6%). A variety of diagnoses included PTEN tumor hamartoma syndrome and CLOVES, amongst others (*n* = 78, 10.8%).

The anatomical locations of treated lesions were the lower extremities (*n* = 226, 33.1%), cervicofacial area (*n* = 172, 25.2%), trunk (*n* = 148, 21.7%), upper extremities (*n* = 95, 12.9%), and multiple locations (*n* = 42, 6.1%).

The vast majority of procedures were performed under general anesthesia (*n* = 1315 (97.0%). Intravenous sedation was utilized in 29 procedures (2.1%). Only 11 (0.1%) procedures were performed with local anesthesia. Data were missing in 67 procedures.

### 3.1. EPO for Preparing Sclerosing and Embolic Agents

In 1384 procedures (96%), EPO was used to prepare sclerosing and embolic agents (Table 1). EPO was used with a single agent in 829 (58.3%) procedures. The most used agents were STS (*n* = 495, 34.8%), followed by ethanol and NBCA. The mean volume of EPO used was 3.85 mL (range: 0.1–25 mL) per procedure. The mean patient weight, available in 537 procedures (39%), was 45.9 kg (range: 3.7–122.6 kg) with a mean weight-adjusted dose of 0.118 mL/kg (range: 0.001–1.73 mL/kg).

### 3.2. EPO as a Contrast Agent

In 56 procedures (4%), EPO was used as the contrast agent for diagnostic lymphangiography. The mean volume of EPO used was 4.8 mL (range: 0.3–13 mL) per procedure. The mean patient weight, available in 36 (67%) procedures, was 27.4 kg (range: 2.4–79.3 kg) with a mean weight-adjusted dose of 0.2 mL/kg (range: 0.04–0.54 mL/kg).

### 3.3. Other Agents

Several embolic and sclerosing agents were used independently of EPO in 249 (17.5%) procedures (Table 2). In addition, opacifying agents were used in 16 procedures (1.1%), including tungsten powder added to NBCA (*n* = 12) and metrizamide powder added to ethanol (*n* = 2) as well as 1% isosulfan blue (Lymphazurin, Tyco Healthcare; Mansfield, MA) (*n* = 1) and methylene blue (Akorn Pharmaceuticals, Lake Forest, IL) (*n* = 1) prior to the introduction of intranodal lymphangiography. Endovenous laser treatment of anomalous veins was additionally performed in 27 procedures (1.9%).

### 3.4. Complications

Procedure-related complications: there were 25 procedure-related complications in 1442 (1.8%) interventions noted in the immediate post-procedural period (Table 3). A total of 18 (1.5%) complications occurred in 1162 sclerotherapy and 7 (3.4%) in 203 embolization procedures. No complications occurred during lymphangiography. A total of 80% (*n* = 5) of complications were minor, including 12 (48%) type A (requiring no therapy and no consequences) and 8 (32%) type B complications (requiring nominal therapy, no consequence; observation overnight). A total of 20% (*n* = 5) of complications were major, including 3 (12%) type C (therapy, minor hospitalization < 48 h), 1 (4%) type D (major therapy, increased care level, hospitalization > 48 h), and 1 (4%) type E (permanent adverse sequela) complications. No death occurred.

The migration of embolic agents, primarily involving glue, into normal veins or pulmonary arteries occurred in 13 (52%) procedures (9 type A, 2 type B, 1 type C, and 1 type D complication). Skin injury and ulceration occurred in 6 (24%) procedures (1 type A and 5 type B complications). Migration into systemic arteries occurred in 4 (16%) procedures (3 type B, 1 type C, and 1 Type E complications). Two (8%) other type A complications noted were myoclonic jerks and acute atrial fibrillation (when a combination of EPO, STS, and ETOH was used in a two-year-old girl with a large venous malformation).

Adverse events not directly related to the procedure and agents were primarily anesthesia-related (e.g., difficult intubation, pulmonary edema after extubating with a need for reintubation, hypoventilation after extubating, etc.) and occurred in 8 (0.6%) interventions.

No adverse reactions related to the use of EPO were noted in the immediate post-procedural period. No allergic reaction was noted, even with subsequent use of EPO.

## 4. Discussion

EPO is used for various diagnostic and therapeutic purposes, such as chylothorax, chylous ascites, chyluria, and peripheral lymphatic fistula or lymphoceles, amongst others. Pieper et al. describe a therapeutic effect of 51–100% when used in lymphangiography in lymphatic leakage [29].

EPO is also widely used in interventional oncology, such as in transarterial chemoembolization (TACE) or as a radiopaque drug delivery vector [30,31], and can also be used as a fiducial marker for radiation therapy of hepatocellular carcinoma [32]. Lencioni et al. reviewed the efficacy and safety data on transarterial chemoembolization (TACE) using lipiodol-based regimens in the treatment of hepatocellular carcinoma (HCC) [33]. They found a high percentage of adverse events (21,461 in 15,351 patients), with liver enzyme abnormalities being the most observed AE, but these patients can hardly be compared to the patients in our cohort. We did not investigate long-term safety in this cohort, as it must be noted that EPO can persist for years, possibly causing cross-allergies [34].

EPO is also used to prepare sclerosing agents or embolics for the treatment of vascular anomalies. Uller et al. gave evidence that preoperative embolization of venous malformations using NBCA with EPO as contrast agent was safe and effective in children, with the potential for minimizing blood loss and inpatient stay [35].

There are many possible complications that can be related to EPO, used either as a single diagnostic agent or in combination with therapeutic agents. Administering the smallest possible volume of EPO is recommended to reduce the risk of EPO-extravasation, or worse, embolization, particularly in children [3]. The user should follow general rules in the usage of EPO. Dosage recommendations based on the manufacturer are given in the “Materials and Methods” section. The route of EPO is easily visualizable under fluoroscopy, but lymphangiography is a time-intense procedure, as the EPO flows very slowly in the lymphatic channels. Use of EPO should be avoided in patients with a history of sensitivity to other iodinated contrast agents because of an increased risk of a hypersensitivity reaction to Lipiodol [36,37,38]. Hypersensitivity reactions to Lipiodol can occur within half an hour and up to several days after administration, although as mentioned, none occurred in this cohort.

Nevertheless, the dose of EPO in the pediatric population has not been clearly defined for various indications. For lymphangiography, maximal weight-based dosing of EPO 0.25 mL/kg has been recommended [25,39]. A similar maximal dose of 0.25 mL/kg (or up to 20 mL) was recommended in adults to reduce the risk of pulmonary embolism when EPO is used for embolization of tumors [40]. Patients in this cohort safely received EPO within this range.

The incidence of complications and occurrence of allergic reactions in procedures with EPO is unknown. To our knowledge, no data exist on the incidence of complications or allergic reactions when EPO is used as a single agent or in combination for diagnosis and/or treatment of vascular anomalies. There are no directly comparable data on this subject. The available studies focus on the treatment success of vascular anomalies (using EPO as a contrast agent and the numbers in these papers are often small [41]). Other studies focused on patient satisfaction after percutaneous sclerotherapy for spongiform venous malformations, showing low complication rates after discharge with an improvement of symptoms, but often needing multiple treatment sessions, as also seen in this study [42]. A recent study by Bagga et al., focusing on “Clinicoradiologic predictors of sclerotherapy response in low-flow vascular malformations”, showed similar low complication rates (11 complications in 1032 sessions) with only minor complications in patients that were treated by sclerotherapy [43].

We collected any complications that occurred during the peri-interventional period (including complications that were not directly related to the EPO and agents used and are most likely anesthesia-related) and gave numbers on complications that were truly procedure-related, i.e., to the therapeutics used. It must be clearly differentiated between expected “complications” and procedural-related complications. We found that there are only very few major complications but that these are truly procedural-related complications. The D/E complications were related to the use of NBCA in embolizations; the C complications were related to STS in venous malformations. The vast majority of complications are minor without any lasting effect. When EPO was used as a single agent, mainly in intranodal lymphangiography, only minor local events, likely due to extravasation, occurred. Complication rates were not higher when EPO was used in combination and were generally low. No allergic reactions occurred even when EPO was used repeatedly and in intervals.

## 5. Conclusions

The use of EPO in children and adolescents for diagnostic or therapeutic purposes is safe and well-tolerated with minimal morbidity. Severe procedure-related complications are rare and primarily caused by the embolic or sclerosing agent. No allergic reactions were noted.

## Figures and Tables

**Table 1:** Combinations and volumes of EPO and main agents.

Agents	Procedures (*n*, %)	EPO (mL)	STS (mL)	ETOH (mL)	Glue (mL)
EPO	56 (4)	4.8 ± 4.1	0	0	0
EPO, STS	515 (36%)	2.8 ± 2.7	8.2 ± 7.1	0	0
EPO, ETOH	213 (14.8)	5.3 ± 3.8	0	21.7 ± 18.3	0
EPO, GL	124 (8.7)	3.7 ± 4.1	0	0	1.0 ± 0.9
EPO, STS, ETOH	257 (17.9)	4.8 ± 3.0	9.0 ± 6.6	16.4 ± 27.6	
EPO, GL + STS	121 (8.4)	7.3 ± 6.6	8.3 ± 7.5	0	1.5 ± 1.2
EPO, GL, ETOH	80 (8.5)	8.3 ± 8.5	0	18.6 ± 16.6	1.7 ± 1.5
EPO, GL, STS, ETOH	55 (3.9)	9.3 ± 5.7	11.3 ± 10.5	15.8 ± 16.1	1.5 ± 0.9

EPO: ethiodized poppy seed oil, STS: sodium tetradecyl sulfate, ETOH: ethanol.

**Table 2:** Additional agents not combined with EPO.

Agent	Use	# of Procedures (%)
Coils ^1^	Embolic	98 (7)
Doxycycline ^2^	Sclerosant	51 (4)
5% ethanolamine oleate ^3^	Sclerosant	46 (3)
Bleomycin ^4^	Sclerosant	32 (2)
Polyvinyl alcoholparticles ^5^	Embolic	16 (1)
Stainless steel, movable-core guidewires ^6^	Embolic	3 (<1)
Recombinant thrombin ^7^	Embolic	3 (<1)
Amplatzer vascular plug ^8^	Embolic	1 (<1)
OK-432 ^9^	Sclerosant	1 (<1)
Onyx ^10^	Embolic	1 (<1)
Gelatin sponge slurry ^11^	Embolic	1 (<1)
Total		253 (18%)

EPO: ethiodized poppy seed oil. ^1^ Cook Medical, Bloomington, IN, USA; ^2^ Vibramycin, Pfizer, USA; ^3^ QOL Medical, Vero Beach, FL, USA; ^4^ APP Pharmaceuticals, Schaumburg, IL, USA; ^5^ Contour SE, Boston Scientific, Natick, MA, USA; ^6^ Cook Medical, Bloomington, IN, USA; ^7^ Recothrom, Baxter Deerfield, IL, USA; ^8^ St. Jude Medical, Minnesota, USA; ^9^ Picibanil; Chugai Pharmaceutical, Tokyo, Japan, ^10^ ethylene vinyl alcohol copolymer embolic agent Onyx, Micro Therapeutics, Irvine, CA; ^11^ Gelfoam; Pfizer, New York, NY, USA.

**Table 3:** Procedure-related complications per type.

Procedure	# of Procedures	Complications	Minor (A–B)	Major (C–E)
Sclerotherapy	1162	18	15	3
Embolization	203	7	5	2
Lymphangiography	56	0	0	0
Total	1422	25	20	5

## Data Availability

The data presented in this study are available on request from the corresponding author.
